# Medical Gymnastics, Including the Schott (Nauheim) Movements

**Published:** 1901-03

**Authors:** 


**Medical Gymnastics, including the Schott (Nauheim) Movements.** By Axel V. Grafstrom, M.D. Pp. 139. London: The Scientific Press, Ltd. 1899.

This will be found a useful book, as it gives a good account of the subject, and is not too long. It deals with massage as well as with gymnastic movements, and the directions given for carrying out these procedures are clear and concise. The sections on movements and medical gymnastics are perhaps the best. The field of usefulness for this mode of treatment is legitimately a large one, the author's views as to the diseases in which it is efficacious are sound, and he does not seek to employ it indiscriminately. Many medical practitioners who recognise the value of medical gymnastics and massage will find the descriptions of the movements to be regularly employed for the relief of various conditions extremely useful.

